# Antibiotic Prescription Trends in Dentistry: A Descriptive Study Using Japan's National Database

**DOI:** 10.1111/jphd.12663

**Published:** 2025-01-23

**Authors:** Kaho Hirayama, Naoki Kanda, Hideki Hashimoto, Hiromasa Yoshimoto, Kazuo Goda, Naohiro Mitsutake, Shuji Hatakeyama

**Affiliations:** ^1^ Division of General Medicine Jichi Medical University Tochigi Japan; ^2^ Department of Emergency and Critical Care Medicine Hitachi General Hospital Ibaraki Japan; ^3^ Institute for Health Economics and Policy Tokyo Japan; ^4^ Institute of Industrial Science The University of Tokyo Tokyo Japan; ^5^ Division of Infectious Diseases, Department of Infection and Immunity Jichi Medical University Tochigi Japan

**Keywords:** antimicrobial stewardship, dentistry, dentists, national administrative claims database

## Abstract

**Objectives:**

Antibiotic prescription trends in dentistry in Japan remain underexplored. This study aimed to describe these trends and evaluate the impact of the national antimicrobial stewardship program launched in 2016.

**Methods:**

Using Japan's national administrative claims database from fiscal year (FY) 2015 to FY 2020, this cross‐sectional study comprehensively analyzed trends in antibiotic prescription by dentists. Prescription rates were computed per 1000 inhabitants yearly and standardized to the FY 2015 national population. Changes in prescription rates were evaluated using Poisson regression analysis.

**Results:**

In FY 2020, the total number of antibiotic prescriptions was 134.4 per 1000 inhabitants per year, showing a 6% decline over the 6‐year period (95% confidence interval, 6%–6%). Third‐generation cephalosporins constituted 52.3% of dental antibiotic prescriptions in FY 2020, though the proportion had slightly decreased. In hospitals, prescriptions of third‐generation cephalosporins decreased from 64.9% in 2015 to 20.3% in 2020, being replaced by penicillin (from 15.0% to 64.0%). However, in clinics, the magnitude of the change was small (third‐generation cephalosporins, 60.5%–53.1%; penicillin, 10.2%–22.2%).

**Conclusions:**

Third‐generation cephalosporins continue to dominate dental antibiotic prescriptions. The increase in penicillin use was much more pronounced in hospitals than in clinics. Strengthening antimicrobial stewardship, particularly in clinics where the majority of dental care is provided, is crucial.

## 1 Introduction

Antimicrobial resistance (AMR) poses a significant global public health challenge. Inappropriate antibiotic usage could usher in an era where these medications lose their effectiveness in treating or preventing infections. Recognizing the gravity of this issue, the World Health Organization (WHO) introduced the Global Action Plan in 2015 to address and mitigate the rise of AMR [1]. Subsequently, in 2016, the Japanese government endorsed the National Action Plan on Antimicrobial Resistance. This plan aimed to reduce the use of oral cephalosporins, macrolides, and quinolones by 50%, and all antibiotics by two‐thirds by 2020 [2].

In Japan, a significant concern revolves around the excessive prescription of third‐generation oral cephalosporins [3, 4]. We previously reported that approximately 56% of oral antibiotic prescriptions by physicians in outpatient settings were directed toward infections for which antibiotics are generally not indicated [3]. Third‐generation oral cephalosporins accounted for 36.9% of all antibiotics prescriptions between 2012 and 2015 [3]. Generally, the use of third‐generation cephalosporins is not recommended for either the treatment or prophylaxis of odontogenic infections [5]. Our recent findings revealed that third‐generation cephalosporins were also prevalent in dentistry, constituting approximately half of all prescribed antibiotics, as indicated by regional claims data [6]. Additionally, a study by Sato et al. [7] using a social health insurance claims database reported that third‐generation cephalosporins for tooth extraction represented 55.3% of prescriptions from 2017 to 2018. These studies had limitations in terms of generalizability, as they were conducted in a single prefecture or used data from a limited population (beneficiaries of a specific insurance system).

In the present study, we conducted a cross‐sectional study using a national claims database in Japan to describe trends in antibiotic prescriptions by dentists.

## 2 Methods

### 2.1 Data Sources and Health Insurance System in Japan

This sequential cross‐sectional study utilized data from the National Database of Health Insurance Claims and Specific Health Checkups of Japan (NDB). The NDB is a nationwide administrative claims database constructed by the Ministry of Health, Labor, and Welfare of Japan. This database contains claims data for all Japanese citizens eligible for insurance programs and the public assistance system. In Japan, three insurance programs provide universal coverage for citizens—the national health insurance system (for self‐employed individuals, unemployed individuals below the age of 75 years, and their dependents), the employee health insurance system (for employees below the age of 75 years and their dependents), and the late elders' health insurance system (for individuals aged 75 years and older). The government fully covers the medical expenses of individuals in financial distress within the framework of a public assistance system.

The NDB includes medical, dental, and pharmaceutical claims, and all claims can be connected by unique, unidentifiable numbers. Information such as sex, age, medical/dental procedure codes with dates of diagnoses, drug codes with dates of prescriptions, and the prefecture where the medical facility is located are all included. Nonelectronic claims are not included in the NDB.

### 2.2 Data Preparation and Measures

In this study, we utilized a unique identifier generated through the combination of birth date, sex, and name to link dental, medical, and pharmacy claims in the database. We then analyzed the dental and pharmacy claims data. Specifically, we extracted data on oral antibiotics prescribed by dentists and analyzed dental prescriptions from April 2015 to March 2021, a period during which the proportion of electronic dental receipts in Japan exceeded 95% [8]. The proportion of non‐electronic claims was 3.6% in fiscal year (FY) 2020 [9]. According to 2022 statistics from Japan, 85.7% of the 105,267 dentists work in clinics rather than hospitals [10].

The antimicrobials were classified according to the World Health Organization Anatomical Therapeutic Chemical (WHO‐ATC) Classification System [11]. In cases where classes were not defined by the WHO‐ATC system, we referred to the classification scheme offered by the AMR Clinical Reference Center in Japan [12]. Antibiotics were categorized into the following groups: tetracycline (J01A), penicillins (J01C), first‐generation cephalosporins (J01DB), second‐generation cephalosporins (J01DC), third‐generation cephalosporins (J01DD), carbapenems (J01DH, J01DI), sulfonamides and trimethoprim (J01E), macrolides (J01FA), lincosamides (J01FF), and quinolones (J01M). In cases where the same antibiotic was prescribed more than once a day, it was counted as a single prescription. Conversely, in cases where different drugs were prescribed on the same day, each drug was counted separately.

### 2.3 Data Analysis

The study initially analyzed national trends in annual antibiotic prescriptions by dentists from April 2015 to March 2021. Prescription rates were described as the number of prescriptions per 1000 inhabitants per year, based on the metric used in previous studies [13, 14]. Subsequently, we described the trends in antimicrobial prescriptions in Japan, stratified by prefecture and type of dental facility (hospital or clinic).

### 2.4 Statistical Analysis

All analyses were performed during fiscal years. To ensure individual privacy, we implemented data masking for groups with fewer than 10 individuals, following the usage regulations of the NDB. The annual prescription rates were standardized using the national population in 2015 as the reference standard for the following steps [15]: First, we tabulated antibiotic prescriptions, stratified by sex and age group and calculated prescription rates in each stratum by year. Subsequently, we standardized the age and sex distribution to that of Japan in 2015, calculating the annual number of prescriptions per 1000 inhabitants.

The 6‐year changes in antibiotic prescription rates per 1000 inhabitants per year were estimated as the ratio of the rate in FY 2015 to that in FY 2020 with its confidence interval (CI). This estimation was performed using a generalized linear regression analysis with a Poisson distribution. To examine the impact of the COVID‐19 pandemic, we conducted an additional regression analysis excluding FY 2020.

All statistical analyses were performed using two‐tailed hypothesis testing at a 5% significance level. The R software (version 4.1.2; R Foundation for Statistical Computing, Vienna, Austria) was used for all statistical analyses.

## 3 Results

### 3.1 Annual Trends in Antibiotic Prescription Ratios by Dentists

**FIGURE 1:**
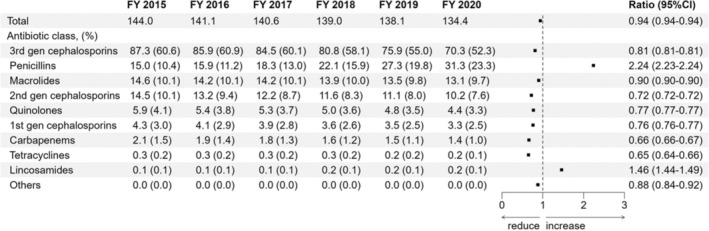
Annual trends of antimicrobial use by dentists in Japan. Consumption is represented by the number of prescriptions per 1000 inhabitants per year, standardized to the national population in 2015 as a reference standard. The change over 6 years was estimated as the ratio of the prescription rates in FY 2020 to those in FY 2015, with 95% confidence intervals, using Poisson regression. Prescription rates refer to the number of prescriptions per 1000 inhabitants per year. 1st gen, first generation; 2nd gen, second generation; 3rd gen, third generation; FY, fiscal year.

Figure 1 illustrates the annual trends in the number of antibiotic prescriptions per 1000 inhabitants prescribed by dentists from FY 2015 to FY 2020. Over this period, the annual prescription rates per 1000 inhabitants were 144.0, 141.1, 140.6, 139.0, 138.1, and 134.4, respectively. The regression analysis revealed a 6% decline (95% CI, 6%–6%) in annual prescriptions over the 6‐year period. Notably, third‐generation cephalosporins emerged as the most frequently prescribed dental antibiotics, constituting 60.6% and 52.3% of all oral antibiotic prescriptions in FY 2015 and FY 2020, respectively. Penicillin ranked as the second most prescribed antibiotic in FY 2020, followed by macrolides, second‐generation cephalosporins, and quinolones. During the 6 years, the prescription rates of third‐generation cephalosporins, macrolides, second‐generation cephalosporins, and quinolones decreased by 19% (95% CI, 19%–19%), 10% (10%–10%), 28% (28%–28%), and 23% (23%–23%), respectively. In contrast, penicillin use increased by 124% (123%–124%). Despite this considerable rise, penicillin constituted only 23.3% of all oral antibiotics prescribed by dentists in FY 2020. An additional regression analysis excluding FY 2020, which was strongly affected by the onset of the COVID‐19 pandemic, yielded results similar to those of the main analysis. The number of antibiotic prescriptions decreased by 1.0% per year (0.990 times per year) in the main analysis from FY 2015 to FY 2020, whereas the decrease was 1.2% per year (0.988 times per year) from FY 2015 to FY 2019.

Table 1 presents antibiotic prescription rates stratified by age and sex. Notably, individuals aged ≥ 65 years exhibited higher prescription rates, regardless of sex. Among men, the prescription rate remained consistent between the age groups of 65–74 and ≥ 75 years (196.0 prescriptions per 1000 inhabitants aged 65–74 years and 200.0 prescriptions per 1000 inhabitants aged ≥ 75 years in FY 2020). In contrast, among women, the rate was lower for those aged ≥ 75 years (155.4 prescriptions) compared to those aged 65–74 years (189.3 prescriptions).

**TABLE 1:** Adjusted incidences of dental antibiotic prescriptions per 1000 inhabitants per year stratified by age and sex.

	FY 2015		FY 2016		FY 2017		FY 2018		FY 2019		FY 2020	
	Male	Female	Male	Female	Male	Female	Male	Female	Male	Female	Male	Female
0–4 years	8.8	6.4	8.2	5.8	7.6	5.4	7.1	5.1	6.4	4.7	6.2	4.3
5–9 years	65.3	53.8	62.1	51.7	60.8	50.3	59.6	48.8	57.2	47.7	52.8	44.8
10–19 years	36.1	40.2	35.0	39.6	34.2	39.0	34.2	38.9	34.0	39.2	33.4	39.6
20–64 years	149.7	157.2	146.9	154.6	146.0	154.5	142.7	152.7	141.0	151.1	138.6	147.2
65–74 years	219.2	208.2	209.8	199.8	206.9	198.4	201.8	196.3	201.8	197.4	196.0	189.3
≥ 75 years	204.2	153.8	203.2	154.7	204.6	157.2	206.7	161.3	205.4	161.9	200.0	155.4

Abbreviation: FY, fiscal year.

### 3.2 Comparison of Annual Trends in Antibiotic Prescription Between Dental Clinics and Hospitals

**FIGURE 2:**
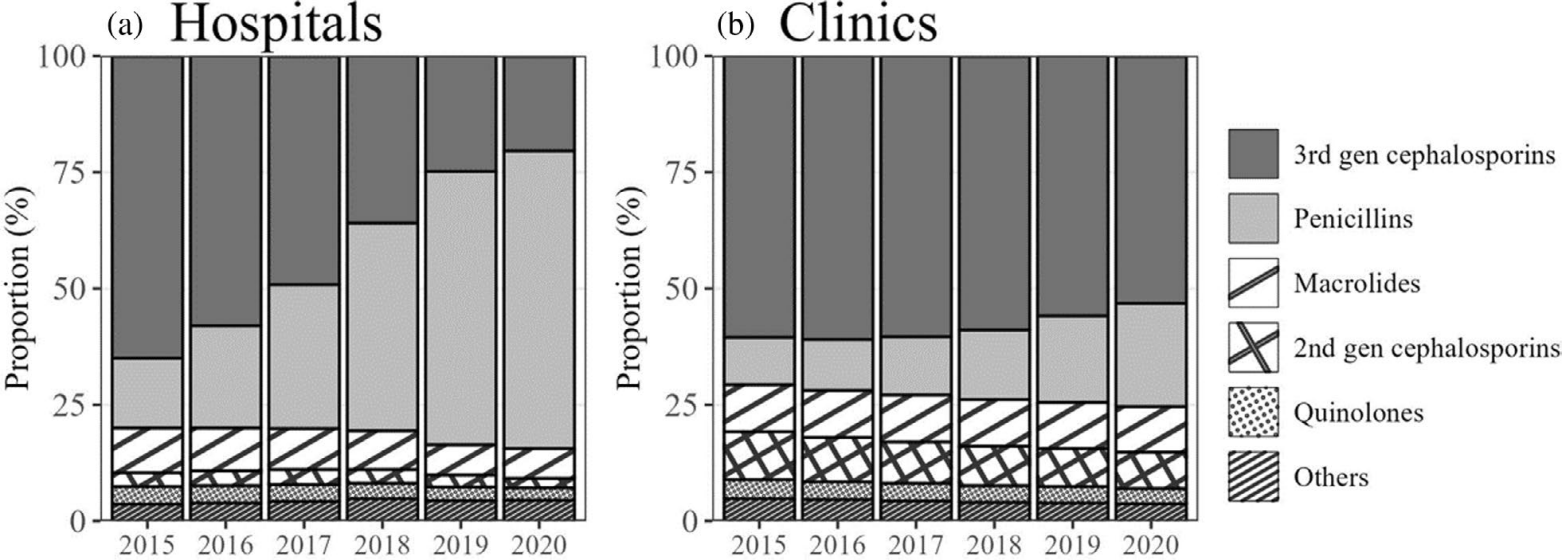
Annual trends in the proportions of antibiotics prescribed by dentists, stratified by the type of facility (hospitals or clinics). Data were aggregated by fiscal year. (a) Hospitals. (b) Dental clinics. 2nd gen, second generation; 3rd gen, third generation.

Approximately 98% of dental antibiotic prescriptions originated from dental clinics. In FY 2020, the prescription rates were 131.0 and 3.4 per 1000 inhabitants in dental clinics and hospitals, respectively. The rates for oral third‐generation cephalosporins prescriptions were 69.6 and 0.7 per 1000 inhabitants in dental clinics and hospitals, whereas those for penicillins were 29.1 and 2.2 per 1000 inhabitants in dental clinics and hospitals, respectively. The proportions of prescriptions by antibiotic class in dental clinics and hospitals are shown in Figure 2. Over the 6‐year period, a notable shift in prescription patterns was noted. In hospitals, the proportion of third‐generation cephalosporin prescriptions markedly decreased from 64.9% in FY 2015 to 20.3% in FY 2020, whereas penicillin prescriptions witnessed a substantial increase from 15.0% in FY 2015 to 64.0% in FY 2020. A similar trend was observed in clinics. However, the magnitude of change was smaller; the proportion of third‐generation cephalosporin prescriptions decreased from 60.5% to 53.1%, and penicillin prescription rates increased from 10.2% to 22.2% between FY 2015 and FY 2020. In hospitals, regression analysis demonstrated a 74% reduction (95% CI, 73%–74%) in third‐generation cephalosporin prescriptions, coupled with a 236% increase (234%–238%) in penicillin prescriptions. In dental clinics, third‐generation cephalosporin prescriptions decreased by 17% (17%–17%), while penicillin prescriptions increased by 117% (117%–117%).

### 3.3 Comparison by Prefecture in Japan

**FIGURE 3:**
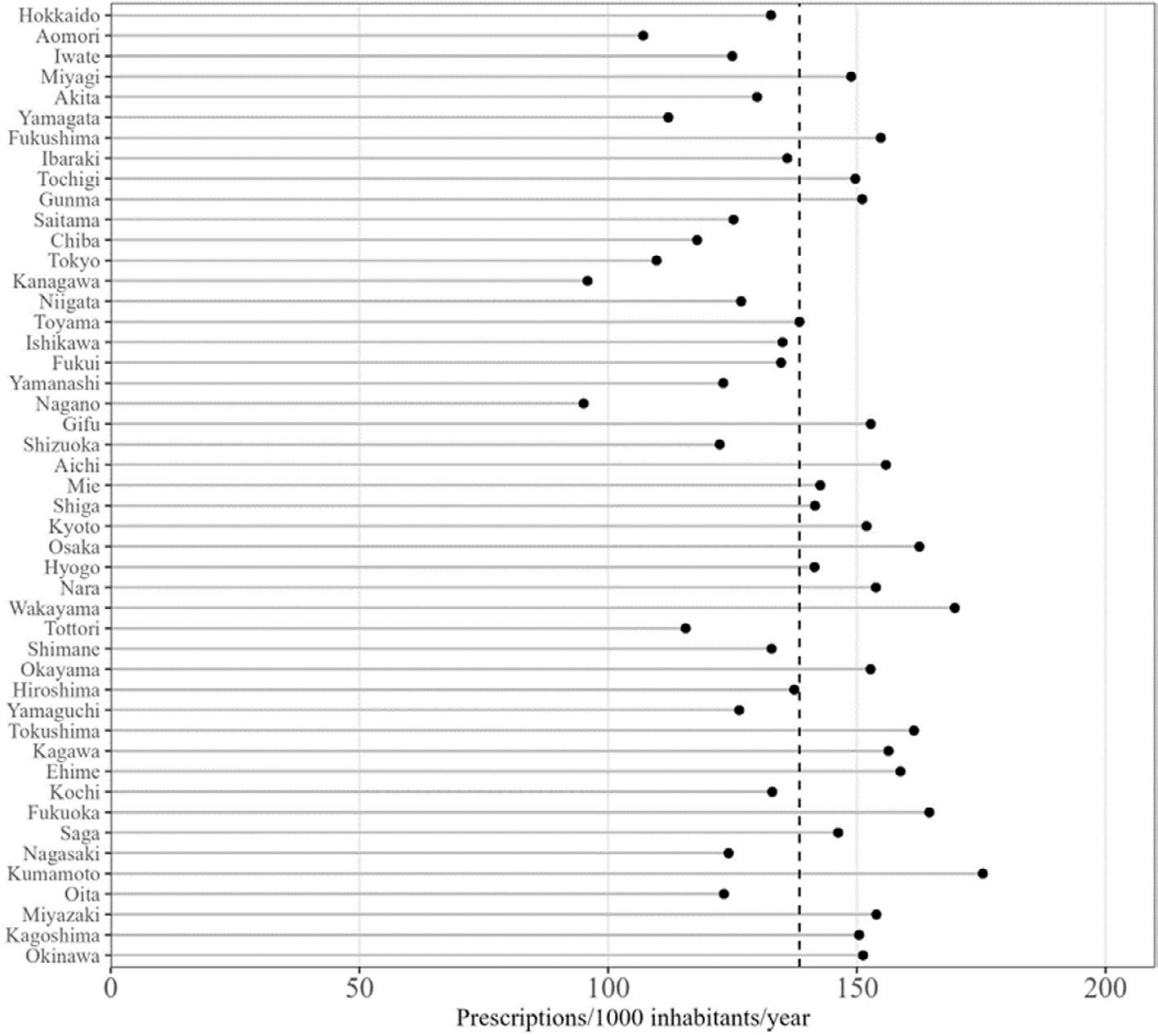
Distribution of the number of antibiotic prescriptions in dentistry by prefectures in Japan in FY 2020. Prescription rates were standardized using the national population in 2015 as a reference standard.

In FY 2020, significant variation in antibiotic prescriptions across prefectures in Japan was observed (Figure 3). The median antibiotic prescription rate was 138.5 (interquartile range: 125.0–151.9) per 1000 inhabitants per year. Notably, Kumamoto, the prefecture with the highest prescription rate, recorded 175.3 prescriptions per 1000 inhabitants in FY 2020, which was 1.84 times higher than that of the prefecture with the lowest prescription rate. Following Kumamoto were Wakayama (169.7 prescriptions) and Fukuoka (164.6 prescriptions) as the top three prefectures with the highest prescription rates. Nagano had the lowest prescription rate at 95.1 prescriptions, followed by Kanagawa (95.9 prescriptions) and Aomori (107.0 prescriptions).

## 4 Discussion

The present study highlighted third‐generation cephalosporins as the most frequently prescribed antibiotics in dentistry during FY 2020, despite a decreasing trend in their prescription rates. A significant shift was observed in hospitals, where third‐generation cephalosporins were being replaced by penicillin. Conversely, dental clinics still predominantly favored third‐generation cephalosporins in their prescriptions.

The widespread use of third‐generation cephalosporins aligns with earlier studies on antimicrobial use among Japanese dentists, specifically using the NDB between 2015 and 2017 [16]. Comparatively, many countries often prescribe penicillins as the most frequently used antibiotics in dentistry. For instance, in England, amoxicillin, macrolides, and cephalosporins constituted 64.8%, 4.5%, and 0.4% of all oral antibiotics prescribed by dentists, respectively [17]. Similarly, in the United States (US), amoxicillin (68.9%) and clindamycin (13.8%) were the most frequently administered antibiotics [18]. Penicillin was the most widely used antibiotic in dentistry across Wales, Norway, Canada, and Australia [19, 20, 21, 22]. In Japan, besides the widespread use of third‐generation cephalosporins, macrolides accounted for approximately 10% of antibiotic prescriptions in dentistry between FY 2015 and FY 2020, despite not being recommended for first‐line use in the guideline [5]. Macrolides and clindamycin are only suggested as alternatives for patients with penicillin allergies [5]; therefore, prescribing macrolides remains more common in Japan than in other countries.

Although the National Action Plan on AMR (2016–2020) aimed for a 50% reduction in oral antibiotic prescriptions by 2020; however, this goal was not achieved. The subsequent 2023 plan proposed a 40% reduction in oral third‐generation cephalosporin prescriptions and a 25% reduction in oral macrolide prescriptions [23]. Therefore, promoting antimicrobial stewardship in cephalosporin and macrolide usage remains crucial in dental services. The proportion of penicillin prescriptions among other antibiotics, which are recommended as the first‐line agents in dentistry, modestly increased from 10.4% to 23.3% between FY 2015 and FY 2020. The growth in this ratio, considered to be an effect of the national antimicrobial stewardship program, was much more pronounced in hospitals than in clinics.

In Japan, the majority of dentists work in clinics, where most patients receive dental care. The extremely low number of antibiotic prescriptions per 1000 inhabitants in hospitals than in clinics is likely due to the lower patient volume in hospitals, rather than an exceptionally low number of antibiotic prescriptions per patient in hospitals than in clinics. In hospitals, a significant decrease in third‐generation cephalosporin prescriptions and an increase in penicillin prescriptions were observed. This pattern aligns with a previous administrative claims study in Japan that described a shift in prescription patterns in hospitals from third‐generation cephalosporins (58% in 2015 to 34% in 2018) to penicillin (16%–38%). Meanwhile, there was little change in prescription patterns in dental clinics [7]. Our results further support this trend, indicating that the drastic shift in prescription patterns in hospitals continued into FY 2020. However, in dental clinics, third‐generation cephalosporins accounted for 53.1% of prescriptions in FY 2020, whereas penicillins accounted for only 22.2%. These findings suggest that antimicrobial stewardship programs are more effective in hospitals than in dental clinics. While hospital dentistry may have an advantage in implementing an antimicrobial stewardship team involving multidisciplinary professionals, organizing such a team in dental clinics could be challenging. Despite this, it remains crucial to align with the context and goals of the AMR Action Plan for dentists working in clinics, especially considering that 97.5% of antibiotics are prescribed by dentists in clinics.

In the US and Canada, dental antibiotic prescriptions were notably more prevalent among patients aged 65 years or older, aligning with findings from the present study [21, 24]. Our study indicated minimal disparity in antibiotic prescription rates between men and women, except for those aged 75 years or older, where men exhibited a higher prescription rate than that of women. Notably, in Canada, rates of antibiotic prescription by dentists were comparable for men and women, with 1.59 and 1.60 defined daily doses per 1000 inhabitants per day, respectively [21]. In the US, women were estimated to have slightly higher odds (OR 1.08; 95% CI: 1.02–1.14) of being prescribed antibiotics compared to men [25]. In Japan, our study revealed that the prescription rate of antibiotics among men did not differ between the age groups of 65–74 and ≥ 75 years. However, among women, the prescription rate per 1000 inhabitants decreased in the age group of ≥ 75 years than in the age group of 65–74 years. The reason for the decrease in antibiotic prescription rates among women aged ≥ 75 years is uncertain but may be influenced by the disparity in the distribution of age between men and women in the ≥ 75 years age group. Among the ≥ 75 years age group, the proportion of those aged ≥ 85 years is 28% among men and 38% among women. In the oldest population, where the proportion of women is higher, there is a possibility that the frequency of dental visits may further decrease.

Regional variations in antibiotic prescription rates in dentistry were evident in our study. This rate tended to be higher in Western Japan than in Eastern Japan, with a 1.8‐fold difference between the highest (Kumamoto) and lowest (Nagano) prescription prefectures. Our recent study on antibiotics in Japanese medical practice also reported higher prescription rates in Western Japan, with a 1.4‐fold difference between the highest (Tokushima) and lowest (Hokkaido) prescribing prefectures [3]. Large regional differences in the number of dentists per inhabitant may contribute to these prescription rate variations [26]. Further research is required to determine the association between antibiotic prescription rates and socioeconomic factors.

In Japan, declines in physician visits and the incidence of infectious diseases during the COVID‐19 pandemic have been reported [27, 28]. In the present study, the pandemic had no notable impact on the trend in the number of antibiotic prescriptions by dentists. Declines in the utilization of dental care services during the pandemic have been reported in the United States [29] and Iran [30]. In the United States, dental utilization decreased more sharply than medical utilization. Furthermore, patients received more invasive dental procedures due to delayed treatment during the pandemic [29]. While the impact of COVID‐19 on antibiotic prescription by dentists may have been minimal in Japan, our analysis did not distinguish between prescriptions for prophylaxis during tooth extraction and those for treating of oral infections; therefore, we are unable to assess any changes in the intended purpose of use.

The present study has some limitations. First, duplicate records can occur when using a unique identifier created by combining date of birth, name, and sex. Although the impact of such duplicates is estimated to be minimal in this study, it remains a common issue when using the NBD. Second, we only analyzed oral antibiotics, as intravenous antibiotics account for less than 1% of prescribed antibiotics by dentists. Third, we did not investigate the indications for antibiotic prescriptions, making it challenging to determine the appropriateness of these prescriptions. Additionally, we could not distinguish between prescriptions for therapeutic and prophylactic use. Finally, clinical outcomes, such as treatment response and incidence of infection, were not explored.

This nationwide claims study revealed that, despite a declining trend, third‐generation cephalosporins remained the most commonly prescribed antibiotics in dentistry. Notably, the goal of the National Action Plan on AMR (2016–2020) was not achieved. To address this, further promotion of antimicrobial stewardship is essential to achieve the goals outlined in the next National Action Plan (2023–2027), with a particular emphasis on dentists working in clinics.

## Ethics Statement

This study adhered to the principles outlined in the Declaration of Helsinki and received approval from the Ethics Committee of Jichi Medical University Hospital (approval number 22‐082). Given the retrospective design and use of anonymized data, the requirement for informed consent was waived by the Ethics Committee.

## Conflicts of Interest

The authors declare no conflicts of interest.
